# Perceptions of Executive Decision Makers on Using Social Media in Effective Health Communication: Qualitative Study

**DOI:** 10.2196/69269

**Published:** 2025-05-21

**Authors:** Norah Abdullah Alanazi, Alia Mohammed Almoajel, Shabana Tharkar, Khalid Almutairi, Farha Nazir Ahmad Mohamad, Bader Saud Talak Almatairi

**Affiliations:** 1 Department of Community Health Science College of Applied Medical Science King Saud University Riyadh Saudi Arabia; 2 King Abdullah International Medical Research Center King Saud bin Abdulaziz University for Health Sciences Riyadh Saudi Arabia; 3 Riyadh Second Health Cluster, Ministry of Health Riyadh Saudi Arabia

**Keywords:** social media, policy makers, decision makers, health communication, Saudi Arabia

## Abstract

**Background:**

The burgeoning rise in social media use has revolutionized information dissemination, rendering social media a vital tool for promoting health campaigns and enhancing 2-way health communication between senders and users. Health planners and policy makers consider social media platforms (SMPs) vital for transferring useful health information to the public. However, there are important concerns about the decision makers’ perceptions of the evolving role of social media in health promotion and education campaigns.

**Objective:**

This qualitative study explored how decision makers perceive the role of social media in health promotion and education. We aimed to shed light on strategic efficacy, real-world challenges, and valuable prospects of using social media for health communication.

**Methods:**

We adopted a qualitative research method involving in-depth, semistructured, face-to-face interviews. We included 13 participants from government and private health care sectors in the Al-Qassim region of Saudi Arabia, who were key players and decision makers in health care programs and reforms. Data were recorded, transcribed verbatim, and analyzed using thematic analysis to identify key themes and patterns.

**Results:**

Five main themes were identified: (1) use of social media (frequency, type of content, target audience, purpose of communication), (2) perceptions of decision makers (how social media influences public health behavior), (3) benefits, (4) challenges, and (5) implications for future use. Participants recognized the positive role of SMPs in spreading health information, particularly in health promotion and awareness campaigns. Communication emerged as a key concept, and WhatsApp, X (Twitter), and Facebook were recognized as major platforms for digital health literacy. The participants used these applications extensively for communication with colleagues, patients, and the public, intending discussion, information exchange, and health promotion campaigns. Content inaccuracy and reliability were identified as major challenges. Furthermore, misinformation and social inequalities were identified as barriers to effective communication. Participants suggested that social media influencers play a more effective role in information dissemination than the health care staff. Far-reaching audiences, visually appealing and engaging content using videos and graphics, and assessing campaign effectiveness using metrics, such as views, shares, likes, and comments, were recognized as major benefits of social media. Participants stressed the promising role of social media in the future as technological advancements in eHealth could revolutionize health care.

**Conclusions:**

SMPs play a vital role in sharing information about health-related initiatives. This research highlights the complexities and potential challenges of using social media for health promotion in Saudi Arabia. It emphasizes the need to develop strategies to combat misinformation, address privacy and confidentiality concerns, and ensure compliance with legal and ethical standards. Encouraging communication among key stakeholders, including health promotion experts, government organizations, social media companies, and the general public, can help establish effective guidelines and protocols to overcome the challenges.

## Introduction

Social media platforms (SMPs) are becoming essential to our everyday lives in today’s digitally connected society. With over 3.6 billion current users and an estimated 4.41 billion users worldwide by 2025, the potential for using these platforms for health promotion has become increasingly apparent [1]. Applications such as Facebook, YouTube, and WhatsApp boast more than 2.5 billion users worldwide, positioning them as effective channels for disseminating health awareness campaigns. Generally, content related to health is well received by users, and social media influencers play a significant role in the successful transmission of health information [2]. The interactive nature of social media fosters community building, user engagement, and the worldwide spread of health-related messages [3]. Health promotion efforts can be strategically directed toward specific demographics and tailored to address particular health concerns [4]. Given these advantages, health promotion campaigns are increasingly shifting from traditional mainstream media to SMPs, allowing for a broader reach in the distribution of health information.

According to the Saudi General Authority of Statistics, 91.2% of the Saudi population aged between 12 and 65 years use the internet and smartphones. Facebook and X (Twitter) are the 2 most common SMPs in Saudi Arabia, with almost 24 million subscribers, making them the most widely reachable sources for information dissemination. Hence, these platforms can be considered a vital source for the dissemination of health-related information.

Executive decision makers, such as policy makers, public health officials, and health care professionals, possess considerable influence in shaping health policies and programs. They play a pivotal role in determining how effectively social media can be used to promote health campaigns [4]. Their perceptions are vital in assessing the success of social media as an educational resource for health promotion. Despite acknowledging the potential benefits, policy makers have expressed concerns about the ethical implications of using social media, fearing data privacy and information reliability, trust, and credibility as the main challenges, especially given the prevalence of false information and fake news on social media in the absence of regulatory guidelines [5,6]. Inequalities in accessing social media are another major concern [7]. Exploring these perceptions plays a crucial role in formulating strategies that ensure social media’s benefits are accessible to everyone [8].

Furthermore, health care transformation is a pivotal objective of Saudi Arabia’s Vision 2030 goals. The realization of these objectives is significantly anchored in the adoption of digital technologies. Saudi Arabia is distinguished by one of the highest worldwide rates of social media engagement, with most of the population actively involved on various platforms. This situation presents a significant opportunity to use social media for health promotion initiatives. There is, however, a limited amount of literature on the research question of how policy makers perceive the role of social media in health campaigns. Hence, in-depth qualitative research is essential to explore their perceptions about social, cultural, and individual factors that influence the development of effective health promotion campaigns via social media. This research aimed to perform a comprehensive analysis of how individuals in decision-making positions perceive the use, benefits, and challenges associated with social media use in health promotion campaigns.

## Methods

### Study Design and Participants

In our study, we applied thematic analysis following the 6-step process described by Braun and Clarke [9] to identify the key themes from decision maker interviews. This qualitative study interviewed 13 participants categorized as executive decision makers in the health sector using 9 semistructured questions. In the first step, participants were selected using a purposive sampling method. Inclusion criteria for study participants included having a lead role in policy decision-making processes and having specific knowledge and experience related to the role of social media use in health promotion. Executive decision makers are individuals holding authoritative positions who play an important role in the decision-making process when formulating or modifying effective health policies and programs. The participants included senior executives and their deputies from government and private health institutions of the Al-Qassim region of Saudi Arabia. In-depth interviews are purposeful conversations designed to elicit relevant information from the participants, incorporating a semistructured question format. Face-to-face semistructured interviews were conducted between September and December 2023, each lasting up to 30 minutes.

### Definitions of Variables

The term “social media” encompasses a range of online platforms and technologies that enable users to share content, interact, and engage with one another. Common SMPs include YouTube, Facebook, WhatsApp, X, TikTok, Instagram, and LinkedIn. The variables examined included (1) usage patterns (the degree to which executive decision makers use SMPs for health promotion activities), (2) engagement and communication (the frequency and quality of social media interactions and communications between executive decision makers and the public), and (3) the perception of reach and impact (the perceived effectiveness and influence of health promotion efforts conducted through social media channels).

The term “health promotion” pertains to strategies and actions designed to enhance public health outcomes and increase awareness of health-related issues. The variables included (1) awareness campaigns (the views of executive decision makers on the efficacy of social media in spreading health-related information through awareness initiatives), (2) behavior change interventions (the perceived contribution of social media in fostering behavior change interventions and promoting healthy lifestyle choices among executive decision makers), and (3) stakeholder involvement (the extent to which social media encouraged active participation and engagement of various stakeholders, including the public, health care professionals, and organizations, in health promotion initiatives).

### Data Collection

After sample selection, the second step involved conducting in-depth semistructured interviews. These interviews consisted of a series of predefined questions related to the role of social media in health promotion. However, based on the participants’ responses, the interviewer further explored particular themes or responses. Step 3 involved audio-recording and verbatim transcription of all interviews to ensure no information was missed. After verbatim transcription, step 4 included detailed data analysis. The variables used were (1) knowledge and understanding of the functionalities of social media among executive decision makers; (2) attitudes, beliefs, and preconceptions held by executive decision makers regarding the role and effectiveness of social media in health promotion; and (3) the degree to which executive decision makers perceive social media as an essential tool for effective health promotion strategies.

#### Interview Questions

The 9 questions were as follows:

- What are the perceptions of executive decision makers of the role of social media in health promotion?
- How is social media currently used for health promotion?
- What are the perceived benefits and challenges in using social media for health promotion?
- How do executive decision makers evaluate the effectiveness of social media as a tool for health promotion?
- What strategies do executive decision makers suggest for improving the use of social media for health promotion?
- How have these perceptions and strategies evolved, particularly in the current context?
- Are any specific SMPs perceived as more effective in health promotion? If so, why?
- How do executive decision makers perceive the impact of social media on health promotion compared to traditional methods?
- What role does audience engagement with social media play in the perceived success of health promotion efforts according to executive decision makers?

### Data Analysis and Coding

In step 4, the data analysis process started with a thorough and repeated reading of the interview transcripts to gain an in-depth understanding of the content to uncover patterns and meanings reflecting participants’ perspectives. The data were divided into small units carrying specific meanings in the initial coding phase. For example, codes such as “communication with the public” and “health awareness dissemination” were identified. Subsequently, codes with similar meanings were grouped into larger themes. For instance, codes such as “awareness dissemination” and “public engagement” were categorized under the broader theme of “benefits of social media.” To ensure the accuracy of this categorization, the themes were reviewed against the original transcripts to confirm their alignment with the participants’ viewpoints, thereby enhancing the consistency of the findings.

The themes were then clearly defined and labeled to capture their essence concisely, such as “challenges in using social media” and “the role of influencers in awareness dissemination.” In the final step, the themes were reported with supporting quotations from participants to maintain the connection between the analysis and the raw data.

ATLAS.ti was used in organizing and analyzing the qualitative data systematically and efficiently [10]. Interview transcripts were imported into the software and categorized according to research topics, such as “benefits,” “challenges,” and “future expectations.” The transcripts were then coded using specific labels that reflected the key ideas within the data, such as “information gathering” and “direct communication with the public.” For example, the code “direct communication” was linked to the statement “We use Twitter and Facebook to communicate with the public and answer their health-related questions.”

The software also facilitated the creation of visual networks, illustrating the relationships between codes and themes in our study. These networks provided a comprehensive view of the data analysis, such as the relationship between the theme “challenges” and the subtheme “misinformation.” This feature added depth to the analysis and helped explore connections within the data.

In step 5, to ensure the credibility of the analysis, the coding process was reviewed by multiple researchers to ensure consistency in interpretation. Disagreements were resolved through discussions, ensuring uniformity in classification—an approach commonly known as intercoder reliability. Additionally, validation was performed by sharing the summary findings with the participants and checking for alignment with their experiences and perspectives. The final step involved compiling a report detailing the findings of the study. This report provided insights into decision makers’ perceptions regarding the role of social media in health promotion in Saudi Arabia. Two primary studies were used to support the methodological approach. The first was by Braun and Clarke [9], which outlines the 6 steps in thematic analysis, and the second was by Friese [10], which provides a comprehensive guide for using ATLAS.ti for qualitative data analysis.

### Ethical Considerations

Informed consent was obtained from all participants before conducting interviews. Anonymity was maintained by replacing their names with pseudonyms or codes in the transcriptions. The study was approved by the Regional Ethics Monitoring Office of the National Committee of Bioethics (NCBE; registration number: H-04-Q001).

## Results

### Participant Details

In total, 13 face-to-face interviews were conducted. The description of the 13 participants (n=11, 85%, males; n=2, 15%, females) is provided in Table 1. The participants held different positions, such as director, deputy director, general director, and department head, executing responsibilities including coordinating with health sectors, leading health promotion programs, spreading health awareness, and participating in decision-making for health programs. The experience varied from a few months in the current position to 20 years. Despite the differences in roles and experience, all participants shared a common goal of promoting health and wellness in their respective areas.

**Table 1:** Background information of participants (N=13) from various health care institutions in the Al-Qassim region of Saudi Arabia.

Participant number	Gender	Job title	Professional specialty	Experience	Role	SMPs^a^ used^b^
1	Male	Executive director	Executive director of the Al-Qassim Health Assembly	20 years	Coordinating between health sectors and financial departments Leading health promotion programs	Yes
2	Male	General director	General director of health affairs in the Al Qassim region	—^c^	Supervising health service provision in the region Leading health promotion programs	Yes
3	Male	Deputy director general	Health promotion and health culture change at the Al-Qassim Health Complex	2 months	Linking programs solving department problems Taking a critical goal approach	Yes
4	Male	Head of the department	Department of Health Education and Awareness at the Buraidah Central Hospital	7 months	Spreading health awareness Educating patients	Yes
5	Male	Deputy director	Diabetes and Endocrinology Center in Al-Qassim	3.5 years	Handling administrative matters Solving problems Handling accreditation of the diabetic foot center	Yes
6	Female	Director	Health Promotion and Education Department at the Diabetes and Endocrinology Center in Al-Qassim	8 years	Handling health education and promotion Raising awareness Using different decision-making approaches	Yes
7	Male	Senior staff member	Department of Communication, Public Relations, and Awareness at a specialized hospital in Buraidah	5 years	Handling health education awareness through social media Targeting different age groups	Yes
8	Female	Social worker and director	Institutional communication and health promotion at the heart center in Al-Qassim	—	Spreading health awareness Preventing heart disease Organizing awareness campaigns since 2005	Yes
9	Male	Director	Maternity and Children’s Hospital in Buraidah	25 years	Providing medical help in the health care field Raising awareness Providing educational services Being involved in decision-making for health promotion programs	Yes
10	Male	Consultant and head	Pediatric Endocrinology and Diabetes Department at the Maternity and Children’s Hospital	9 years	Established the department Heading the department Providing specialized care	Yes
11	Male	Director	Al-Badaie Hospital	8 years	Leading the hospital’s quality accreditation Participating in decision-making for health programs	Yes
12	Male	Director	Director of the Al-Mudhnab Hospital, supervisor of the public health sector in the Al-Mudhnab Governorate	8 years	Promoting health awareness Promoting interest in health programs and disease prevention Participating in decision-making	Yes
13	Male	Director	Director of the Buraidah Central Hospital	20 years	Handling the health care sector to promote a health culture Following up on the health program	Yes

^a^SMP: social media platform.

^b^WhatsApp, Snapchat, YouTube, X, Instagram, Google, and Facebook.

^c^Not applicable.

### Themes and Subthemes

The 5 themes and subthemes that emerged from the interviews were (1) the use of social media, (2) positive perceptions, (3) benefits, (4) challenges faced using social media for health promotion activities, and (5) future use of social media. The first theme provided insight into the specific platforms being used, the frequency and types of content being shared, and the target audience being reached. The second theme included user opinions and perceptions related to the level of influence social media has on public health behaviors and the public’s overall engagement with health-related content on these platforms. The third theme identified benefits and positive outcomes of using social media as a health promotion tool, such as increased public awareness, improved access to health information, enhanced communication between health care providers and individuals, and potential cost-effectiveness. The fourth theme included obstacles, limitations, and potential risks associated with the use of social media. The final theme described the future use of social media as a potential and powerful tool for information dissemination. Overall, by identifying these themes, the study aimed to provide a comprehensive understanding of decision makers’ perceptions of the role of social media in health promotion in Saudi Arabia.

#### Theme 1: Use of Social Media

This theme focused on the frequency and purpose of social media use. Most of the participants used it extensively for both personal and professional reasons. Communication emerged as a key concept, with participants using SMPs such as WhatsApp, X, and Facebook to communicate with employees, patients, and the public. Information gathering was another important concept within this theme. Participants expressed interest in using social media to follow research and scientific content. Platforms like X and WhatsApp were mentioned as valuable sources of information for health care decision-makers.

I use social media on a daily basis at work, whether to communicate with employees or with patients for awareness, promotion, and advertising.

Participant 1

I always follow studies and social media, especially the Gora platform, which is similar to Twitter, where questions and answers are useful.

Participant 3

I use social media more professionally than personally...to disseminate health messages, awareness materials, questionnaires, reports, and videos.

Participant 8

Our analysis indicates that social media serves as an essential resource for health care decision makers to interact with the public, share health-related information, and gather pertinent data, enhancing their professional abilities. Gaining insight into the role of social media use can guide the formulation of effective strategies for health promotion activities.

#### Theme 2: Perceptions of Social Media in Health Promotion

The second prominent theme identified from the data pertained to the perceived beneficial influence of social media on health promotion. Participants highlighted the capacity of social media to efficiently spread health-related information and encourage individuals to adopt healthier behaviors. A significant aspect of this theme was the importance of sharing accurate and trustworthy health information on social media for effective behavior change:

It can be used to change individuals’ behavior towards better health, but the information disseminated must be accurate and reliable.

Similarly, another participant emphasized the role of social media in correcting health information and preventing misinformation:

It can be an effective tool to promote public health...and correcting scientific material from reliable platforms.

Another subtheme was the positive impact of social media on health awareness and disease prevention. One participant shared their successful experience with the “Love of Health” account launched by the Saudi Ministry of Health, which had a positive impact by spreading health awareness and disease prevention. Another participant also mentioned an example of raising breast cancer awareness among middle school students, which encouraged their mothers and families to undergo screening.

Participants emphasized the positive impact of social media on health promotion. They believed that the effectiveness of social media in promoting health depends on the culture of the person using it. One participant stated:

Social media can be an effective tool to change individuals’ behavior if it is directed and used in the right direction.

Another participant echoed this sentiment:

It is preferable not for specialists to appear directly on social media, but rather to choose influencers in the field of social media to disseminate health information.

This theme implied that social media has the potential to be a powerful tool in health promotion. It can effectively disseminate accurate health information, correct the circulating misinformation, and raise awareness about important health issues. However, it is important to ensure that the information shared is accurate and reliable. The participants’ belief that influencers are more effective in disseminating health information rather than specialists suggests that the public may respond better to popular and trustworthy individuals. This has implications for health promotion strategies and selecting message bearers on social media.

#### Theme 3: Benefits of Using Social Media for Health Promotion

Participants acknowledged several concepts within this theme. This theme illustrated the potential benefits of using social media to reach a larger and more diverse audience in an appealing and engaging manner, as well as assess the impact of the content by the number of views and likes. This was supported by the following statement:

Social media helps us reach a wider audience beyond our local community.

Participants also reported that SMPs offer opportunities to present health information in a visually appealing and engaging manner. One participant mentioned:

We can use images, videos, and graphics to make the health message more interesting and attractive.

Another significant subtheme was the ability to assess the impact of the message. One of the participants explained:

We can analyze the number of views and interactions with published health content to assess the impact of our campaigns.

Participants cited various methods to measure effectiveness, including analyzing views and interactions, studying the impact on behavior, and using indicators (eg, followers, shares, likes, and comments). Some participants mentioned interacting with the public, communicating with experts, and regularly updating information on social media. Others mentioned using the X platform to share questionnaires and to evaluate their social media use.

Another subtheme underlined additional benefits, such as faster communication, time and cost savings, and enhancement of interaction and cooperation among individuals and communities.

The potential benefits of using social media for health promotion underscore its significant role in enhancing public health. Social media allows for reaching a broad audience, presenting information in engaging ways, and assessing the impact of campaigns. These SMPs can be valuable tools for raising awareness, improving communication, and fostering interaction and collaboration in health promotion initiatives.

#### Theme 4: Challenges in Using Social Media for Health Promotion

Participants expressed concern about the dissemination of inaccurate information. As a participant stated:

Posting inaccurate or misleading information is a major challenge when it comes to using social media for health promotion.

Participants also highlighted the lack of clear standards for health content. A participant noted:

One of the challenges I see is the lack of clear standards for health content. It is important to ensure the credibility and privacy of the information shared.

Another participant mentioned the limitations of reaching some demographic groups via social media. This concept highlighted the inequalities in social media use by certain sections of society. Other challenges, such as the incompatibility of some platforms with the practical environment, the lack of audience interaction as required, and the organization’s exposure to accountability, criticism, or ridicule, were also mentioned.

Additionally, the participants provided examples of strategies to manage these challenging risks:

- Review all materials submitted on social media to ensure their accuracy and appropriateness
- Use various SMPs
- Apply legal and ethical controls
- Form a specialized team in social media management, cooperate with relevant authorities and health influencers, and verify sources and references before publishing information

Participants mentioned that there are legal controls in place to regulate the use of social media in the field of health. However, specific details of these regulations were not provided in the data. Overall, this subtheme highlighted the complexities and potential pitfalls of using social media for health promotion. They also emphasize the need for strategies to tackle misinformation, ensure privacy and confidentiality, and adhere to laws and ethics.

#### Theme 5: Future of Social Media in Health Promotion

**Figure 1:**
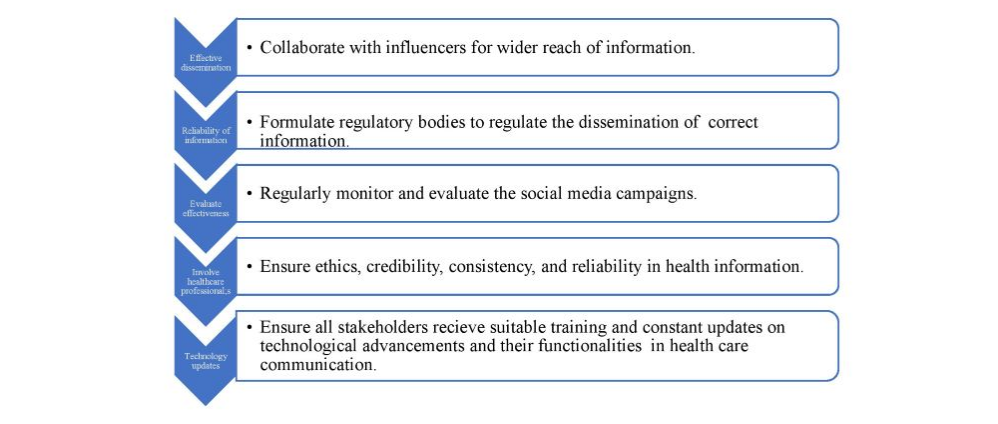
Recommendations for effective use of social media in health communication.

The fifth theme highlighted the importance of the future role of social media in health promotion. Participants expressed optimism about the future, crucial role of social media in health promotion and suggested various strategies for effectively leveraging it in health promotion initiatives. They also stressed the need to establish clear standards for health-related content, increase interaction with followers, collaborate with stakeholders, and provide training for health workers to use social media effectively. In addition, they highlighted the importance of cooperation between different stakeholders to enhance the use of social media in health promotion. They proposed strategies such as constantly updating content, using multimedia, and actively interacting with the audience to effectively use social media for health promotion (Figure 1).

Participants proposed the idea of a social media calendar that interacts with designated days related to health, such as Breast Cancer Day and Walking Day. Additionally, they mentioned the potential usefulness of upcoming interactive tools, such as ChatGPT. They also suggested the potential usefulness of platforms such as TikTok, if developed and used correctly. They expected rapid development of social media in the future and believed it can be beneficial for health if directed in the right way.

One main subtheme was the potential of social media to reach and influence different demographic groups, particularly the youth and teenagers. Participants mentioned that the culture of society is changing, especially among the youth and teenagers, due to social media and that they try to reach these groups through friendship rather than traditional advice. Additionally, they highlighted the need to balance real and virtual life to avoid excessive isolation. They were optimistic about the potential usefulness and fun of upcoming platforms, although they found it difficult to predict specific ones. However, they believed caution must be exercised in reaching out to teenagers solely through social media. One participant stated:

I try to reach them through friendship more than traditional advice because their culture has changed a lot. They prefer to talk to friends rather than listen to advice from adults.

Another subtheme was the potential for social media to bridge the gap between health care professionals and the public. Participants mentioned the benefits of having volunteers who can check and correct concepts, contributing to the promotion of health. One participant emphasized this by saying:

Having volunteers to help with checking and correcting concepts is so important. It contributes to promoting health and ensures that accurate information is being shared.

The participants also discussed the emergence of new technologies and platforms in the future, such as virtual clinics, the virtual hospital, and eHealth programs. These technologies were seen as promising tools for promoting health. One participant described the virtual hospital as a qualitative leap that provides speed in providing services with high quality and efficiency.

## Discussion

### Principal Findings

One of the main findings of the study is the proficient application of social media by health care decision makers to share health-related information. By using platforms such as YouTube and Facebook, executive decision makers can effectively engage a broader audience and create awareness about diverse health topics. Prior research has underscored the efficacy of social media in conveying health information, especially among the younger population [11-14]. In Saudi Arabia, where a considerable segment of the population comprises youths, social media holds significant potential for enhancing health literacy and increasing awareness regarding preventive health measures. Research has consistently shown that SMPs can reach a wide audience and effectively disseminate health-related information [15,16]. SMPs can be used to promote physical activity among young adults. The findings indicated that social media interventions have the potential to positively influence behavior change and improve health outcomes [16].

Another significant finding pertains to the capacity of social media to engage in effective communication. Platforms such as X and Instagram serve as interactive channels that enable direct engagement with beneficiaries, allowing for the addressing of their concerns. X’s real-time updates and concise nature make it an efficient platform for sharing quick updates and engaging with a larger audience. The use of Instagram suggests a preference for visual content, which can be effective in conveying health information in a visually appealing manner. This visually appealing approach can enhance users’ comprehension and retention of health messages. This aligns with earlier studies that have enumerated the role of social media in fostering 2-way communication between health care providers and end users [17]. Such interaction is vital in the realm of health care decision-making, as it equips decision makers with valuable insights into public opinions and preferences, thereby enabling them to make informed decisions aligning with community needs. In addition, virtual communities of individuals sharing common goals and concerns provide a supportive environment for sharing experiences, seeking advice, and forming connections with like-minded individuals, fostering a patient-centered approach to health care [18].

Furthermore, it was recognized that social media influencers are more effective in disseminating health information compared to specialists in their relatability and popularity, which can effectively create a sense of normalization around healthy behaviors and positively influence individuals’ health-related decisions. This finding aligns with previous research that highlights the role of social network dynamics in influencing health behaviors. Studies have shown that individuals are more likely to adopt healthy behaviors when they perceive them to be reliable within their social networks [19]. Furthermore, the preference for influencers over specialists in health promotion highlights the importance of trust and credibility in disseminating health information.

Another important finding of the study is evaluating the effectiveness of health promotion campaigns by tracking metrics. Decision makers appreciated this feature, which tracks the number of views, reactions, shares, and comments as indicators of the end users’ engaging behavior. This information helps policy makers tailor the content according to society’s needs. Many studies support this view of evaluating web-based outcomes with real-world outcomes [20,21]. However, it is crucial to acknowledge the potential challenges and risks involved, as highlighted by the participants. The impact of social media can be negative if the information is incomplete or if the message bearer is not trustworthy. Inaccurate or misleading health information can potentially harm the public by promoting ineffective or even harmful practices. Thus, careful monitoring and evaluation of social media campaigns is necessary to ensure the accuracy, reliability, and ethical nature of the information being shared. Addressing legal and ethical issues is another major challenge. The decision makers emphasized adhering to laws and ethical guidelines while disseminating health-related information on SMPs. These findings are consistent with previous research that accentuated the negative influence of misinformation resulting in anxiety, confusion, harmful practices, and psychological distress [22,23]. The verification of sources emerged as a key challenge identified by the participants. End users’ practice of blind belief in shared content is a major setback in tackling fake information [24]. This challenge highlights the importance of promoting digital literacy and critical appraisal of health content shared on social media. Participants emphasized the importance of cooperation with relevant authorities to address the challenges in using social media in health promotion. This underscores the need for collaboration between health promotion professionals and government entities to establish guidelines and regulations concerning social media use, which aligns well with other studies. Similar findings were reported in a study by Kanchan and Gaidhane [25], who highlighted the significance of collaboration between public health agencies and SMPs to ensure dissemination of accurate and reliable information. This study calls for developing specific regulations governing social media use in the health care sector. Saudi Arabia lacks comprehensive regulations specifically designed for social media health promotion activities. Drawing from international best practices, such as the guidelines established by the World Health Organization and the American Medical Association, can provide a foundation for Saudi Arabia to develop its regulations. These regulations should address key concerns, such as the quality and accuracy of health information, the protection of individual privacy, and the prevention of misleading advertisements and health claims.

Finally, the participants’ opinions of the eHealth concept involving the use of advanced communication technologies in health information dissemination will play an influential role in the future. Many studies support the persuasive role of social media in creating awareness, such as the harm associated with smoking, chronic disease screening, reproductive health, and the most famous COVID-19 prevention campaigns [26,27]. Kelleher and Morino [28] found that social media interventions are able to increase knowledge about reproductive health among young people and positively affect their behaviors. This aligns with the findings of our study, which suggests that social media will continue to play a vital role in promoting health, particularly among the youth population in Saudi Arabia. The implications of these findings go beyond the dissemination of health information. The advancements in social media technologies mentioned by the participants suggest that social media has the potential to bridge the gap between health care professionals and the public and provide efficient and high-quality health care services. This aligns with the eHealth concept, which involves using information and communication technologies, including social media, to improve health care delivery and empower individuals to take control of their health. The findings of this study suggest that social media has the future potential to become an even more effective platform for providing health care services and engaging with the public.

Health care transformation is a key component of Saudi Arabia’s Vision 2030 goals. Facilitating this health care transformation by adopting innovative technologies is crucial for accomplishing the goals. The integration of cutting-edge technologies and the use of interactive social media can enhance the population’s understanding of health and wellness, thereby serving as an important tool for informed decision-making. The commitment to innovation in health care will play a vital role in improving the population’s well-being and advancing the strategic vision for a healthier and more sustainable Saudi Arabia.

### Limitations

The study has certain limitations. By including only executive decision makers in the interviews, the findings may not be generalizable to a broader population. The perspectives of other stakeholders, such as health care professionals, were not incorporated, which could introduce information bias. Additionally, this research was qualitative and relied on in-depth interviews. This could have added bias due to the reflexivity of the researcher because of assumptions or critical thinking influencing the interview process. Although this approach provides rich and detailed insights, it did not provide quantitative measurements of social media’s effectiveness. Future research should include diverse groups of the population, including health care workers and the general public, to provide a more comprehensive view. Future studies could also use quantitative methods to better explore the use of social media in health campaigns.

### Conclusion

In conclusion, this study provided valuable insights into the perceptions of executive decision makers in Saudi Arabia of the role of social media in health promotion. The use of specific platforms reflects preferences and functionalities that can inform the development of targeted strategies for health promotion. The study calls for the development of specific regulations governing the use of social media in the health care sector. These regulations should address key concerns, such as the quality and accuracy of health information, the protection of individual privacy, and the prevention of misleading advertisements and health claims.

The findings of this study shed light on the complexities and potential pitfalls of using social media for health promotion purposes in Saudi Arabia. It highlights the crucial need for strategies to tackle misinformation, ensure privacy and confidentiality, and adhere to laws and ethics and emphasizes enhanced regulatory efforts with collaboration between the government, social media companies, and the public. Facilitating dialogue between key players, such as health promotion professionals, government entities, SMPs, and the public, can initiate the formulation of effective guidelines and protocols to overcome the identified challenges.

## Abbreviation

SMP: social media platform

## Data Availability

The qualitative data recorded, transcribed, and analyzed during this study are available from the corresponding author upon reasonable request.
